# Application of transformer architectures in generative video modeling for neurosurgical education

**DOI:** 10.1007/s11548-024-03266-0

**Published:** 2024-09-13

**Authors:** Aaron Lawson McLean, Felipe Gutiérrez Pineda

**Affiliations:** 1 Department of Neurosurgery, Jena University Hospital – Friedrich Schiller University Jena, Am Klinikum 1, 07747 Jena, Germany; 2 Department of Neurosurgery, School of Medicine, University of Antioquia, Medellin, Colombia

**Keywords:** Neurosurgery training, Generative video models, Surgical simulation, Medical education technology, Ethical considerations in surgical training

## Abstract

**Purpose:**

This article explores the potential impact of OpenAI’s Sora, a generative video modeling technology, on neurosurgical training. It evaluates how such technology could revolutionize the field by providing realistic surgical simulations, thereby enhancing the learning experience and proficiency in complex procedures for neurosurgical trainees.

**Methods:**

The study examines the incorporation of this technology into neurosurgical education by leveraging transformer architecture and processing of video and image data. It involves compiling a neurosurgical procedure dataset for model training, aiming to create accurate, high-fidelity simulations.

**Results:**

Our findings indicate significant potential applications in neurosurgical training, including immersive simulations for skill development and exposure to diverse surgical scenarios. The technology also promises to transform assessment and feedback, introducing a standardized, objective way to measure and improve trainee competencies.

**Conclusion:**

Integrating generative video modeling technology into neurosurgical education marks a progressive step toward enhancing training methodologies. Despite challenges in technical, ethical, and practical domains, continuous development and evaluation could lead to substantial advancements in surgical education, preparing neurosurgeons more effectively for their demanding roles.

## Introduction

The advent of generative video modeling technology, such as Sora, developed by OpenAI (San Francisco, CA, USA), heralds a potential revolution in medical education, particularly within high-stakes, skill-dependent specialties such as neurosurgery [1, 2]. Rooted in text-conditional diffusion models trained on expansive datasets of video and image content, Sora’s technology offers the theoretical capability to simulate realistic surgical scenarios. While the practical application of such technology in neurosurgical training is not yet realized, this manuscript engages in a speculative discussion on how Sora could be meaningfully integrated into neurosurgical education. The article explores the innovative aspects of this technology and its potential to transform training methodologies. It also critically addresses the substantial development and validation that are required before such a system can be implemented. This theoretical exploration aims to illuminate both the potential enhancements Sora might bring to medical training and the significant hurdles that must be overcome to bring such digital simulations to the forefront of educational strategies in neurosurgery.

In the rapidly evolving landscape of medical education technology, the development of generative video modeling tools like Sora illustrates significant advancements yet underscores the nascent state of their application in clinical training environments [1, 3]. While Sora, developed by OpenAI, represents a leading edge in simulation technology with its high-fidelity video capabilities, it is not yet available to the general public [4]. Meanwhile, similar systems such as Luma (Luma AI, San Francisco, CA, USA), Kling (Kuaishou Technology, Beijing, China), Pika (Pika, Palo Alto, CA, USA), and Runway (Runway, New York City, NY, USA) are emerging, each contributing to the spectrum of AI video tools capable of transforming text, images, and videos into dynamic video educational content [5, 6].

These developments indicate a promising future for integrating more sophisticated, AI-driven simulations in medical training, although the full realization of these technologies in practical settings awaits further development and empirical validation [7]. As such, our discussion and proposals remain speculative but are grounded in a realistic anticipation of technology’s trajectory and its potential impact on neurosurgical education.

## Methods

To investigate the integration of generative video modeling technologies into neurosurgical training, we employed a structured approach focusing on the theoretical underpinnings and practical applications of these technologies. This methodology encompasses a comprehensive analysis of transformer architectures and spacetime patch processing techniques, critical to generating realistic surgical simulations. The subsequent analysis draws on a broad spectrum of technological advancements and empirical evidence within the domain of medical education, reflecting on the broader implications and potential of such technologies in reshaping neurosurgical training. This methodology is grounded in the principles outlined by Reeves and Hedberg’s design-based research (DBR) framework, which emphasizes iterative analysis, design, development, and implementation cycles to address complex problems in real-world settings [8].

### Theoretical framework for Sora integration

The integration of Sora and similar generative technologies into neurosurgical education introduces a forward-looking approach to training methodologies, as illustrated in Table 1. Sora utilizes a transformer architecture to process spacetime patches from video and image latent codes, aiming to generate high-fidelity video content [1]. In simpler terms, Sora works by breaking down videos and images into small pieces, much like a puzzle, and then uses a smart system to understand and recreate these pieces into new, high-quality videos.

**Table 1:** Comparative analysis of traditional versus generative video model-based neurosurgical training

Criteria	Traditional training methods	Generative video model-based training (e.g., Sora)
Accessibility	Limited by the availability of cases, surgical schedules, and geographical location	High accessibility through virtual platforms, allowing for remote and asynchronous learning
Range of pathologies	Constrained by the clinical case mix at the training institution	Unlimited, as any pathology could potentially be simulated, including rare and complex cases, enhancing the breadth of training
Learning environment	High stakes and high pressure, with real patient outcomes at stake	Risk-free, allowing for exploration, experimentation, and repetition without consequences to patient safety
Customization and feedback	Generally one-size-fits-all, with feedback dependent on the attending surgeon’s availability	Highly customizable to individual learner’s needs, with potential for immediate, data-driven feedback
Scalability	Scalability is limited by physical and human resources	Highly scalable, capable of serving an unlimited number of trainees simultaneously without additional resource allocation
Ethical and legal concerns	Involves ethical considerations regarding patient consent and the impact of training on outcomes	Raises ethical questions related to the use of patient data for creating simulations and the potential for misuse of generated content
Technical requirements	Relatively low, primarily requiring access to surgical tools and patients	High, requiring advanced computing resources, data storage, and ongoing technical support for the generative video model’s operation
Cost	Varies, generally high due to the need for physical resources and faculty time	Potentially lower over time, after initial investment in technology development and acquisition

This table presents a detailed comparison between traditional neurosurgical training methods and hypothetical generative video model-based training, highlighting key differences in accessibility, training scope, ethical considerations, and technical requirements

In a neurosurgical context, this approach would begin with the collection and annotation of video recordings of neurosurgical procedures, detailing key procedural steps, anatomical landmarks, and decision-making points. Training the Sora model on this enriched dataset is intended to produce simulations that accurately reflect the complex dynamics of neurosurgical operations. The effectiveness of such an integration rests on the continuous advancements in technology and the accumulation of empirical evidence within the medical education domain. Google, Meta, and the startup Runway represent additional entities that have showcased technology akin to Sora, indicating a broader industry trend toward the development of advanced video generation models [9, 10].

### Potential applications in neurosurgical training

The potential integration of Sora and similar technologies into neurosurgical training, as presented in Table 2, can be viewed as a pathway to enriching the educational landscape. It is envisioned to support the acquisition and enhancement of surgical skills via high-resolution simulations, offering trainees a broad spectrum of neurosurgical techniques in a visually immersive and interactive format. Such simulations aim to facilitate skill development within a controlled environment, minimizing risks to patient safety [11].

**Table 2:** Applications and limitations of generative video modeling in neurosurgical training

Application	Description	Limitations
Skill acquisition	Allows for the visualization and repetition of surgical techniques in a controlled environment	May not fully replicate the tactile feedback and variability of real-life surgery
Scenario-based learning	Enables the simulation of complex surgical cases, including emergency scenarios and rare pathologies	Generated scenarios may lack the unpredictability and complexity of actual surgical situations
Assessment and feedback	Facilitates standardized assessments and personalized feedback based on performance in simulated scenarios	Feedback mechanisms may not capture the nuances of surgical judgment and decision-making skills
Cognitive skill development	Enhances diagnostic reasoning, surgical planning, and intraoperative decision-making through simulation	Limited by the model’s ability to accurately simulate the dynamic nature of surgical environments and patient responses
Ethical and legal training	Provides a platform for discussing and understanding the ethical implications of neurosurgical interventions	Ethical scenarios generated by AI may not fully encompass the moral complexities encountered in clinical practice

This table outlines the potential applications of the Sora generative video model in neurosurgical training, along with associated limitations, emphasizing the balance between technological innovation and the need for realistic, comprehensive educational experiences

Moreover, the capacity of Sora to simulate a wide range of surgical scenarios—from common to rare cases—could serve to broaden cognitive skills, encompassing diagnostic reasoning, surgical planning, and intraoperative decision-making (Table 3). This approach seeks to overcome traditional training limitations by providing access to a diverse array of surgical experiences. Research by Reck-Burneo et al. [12] demonstrated that surgical trainees experienced notably increased levels of confidence after viewing operative videos compared to those who studied surgical techniques through peer-reviewed manuscripts. This finding underscores the potential of Sora’s video modeling technology to enhance neurosurgical education by providing dynamic, visual learning experiences that could improve trainee confidence and competence more effectively than traditional text-based methods.

**Table 3:** Specific Sora prompts for neurosurgical training scenarios

Scenario prompt	Objective	Description	Educational focus	Technical challenge
Intracranial tumor Resection	Skill acquisition	A video simulation of the step-by-step process of resecting a brain tumor, including the approach, craniotomy, and tumor removal	Enhancing technical proficiency in tumor resection, understanding tumor anatomy, and surgical approaches	Ensuring realistic tissue textures and tumor behavior during resection
Endoscopic third ventriculostomy (ETV)	Procedure familiarization	Simulation of an ETV procedure for obstructive hydrocephalus, showcasing the endoscopic path and ventriculostomy	Familiarizing with endoscopic equipment, navigation through ventricular system, and decision-making for ventriculostomy site	Accurately modeling fluid dynamics of CSF and the endoscopic view within narrow ventricular pathways
Spinal fusion surgery	Technique mastery	Detailed generation of a spinal fusion procedure, including pedicle screw placement and bone grafting	Mastery of spinal anatomy, screw placement technique, and understanding fusion principles	Replicating the haptic feedback of pedicle screw insertion and bone density variations
Aneurysm clipping	Decision-making enhancement	A complex scenario involving the strategic planning and execution of clipping an intracranial aneurysm	Improving surgical planning, clip selection, and critical decision-making under varying aneurysm presentations	Modeling the delicate manipulation of vascular structures and the aneurysm’s response to clipping
Emergency decompressive craniectomy	Cognitive skills development	Emergency scenario of performing a decompressive craniectomy for acute subdural hematoma with rising intracranial pressure	Developing the ability to quickly diagnose, plan, and execute a lifesaving procedure under time pressure	Simulating the dynamic changes in brain morphology and intracranial pressure in response to hematoma evacuation

This table lists five detailed examples of Sora prompts designed for neurosurgical training, outlining the objectives, descriptions, educational focus, and the technical challenges each scenario aims to address. These prompts highlight the potential of generative video models to enhance various aspects of neurosurgical training, from technical skill acquisition to complex decision-making

Concerning assessment and feedback, the ability of Sora to create standardized surgical scenarios could potentially transform how trainee competencies are evaluated. By generating consistent and objective criteria for assessment, the technology may offer a structured platform for feedback, tailored to the learning trajectories of individual trainees. While the anticipated benefits of such technology are significant, they are contingent upon further development and rigorous evaluation to determine their practicality, effectiveness, and impact on educational outcomes in neurosurgery.

### Strategic implementation of generative video modeling in neurosurgical education: a phased approach

In the development of neurosurgical education, the European training requirements outlined by Whitfield and colleagues [13] provide a robust framework that allows generative video modeling technologies such as Sora to be aligned with current educational standards. The competency-based curriculum described emphasizes a multidimensional approach to neurosurgical education, focusing on entrustable professional activities across various stages of medical training. Moreover, there is an explicit recommendation that “trainees should attend simulation training throughout the training program.” The model proposed by Whitfield et al. aligns with the proposed integration of video modeling technologies by providing structured, competency-driven benchmarks that Sora simulations could aim to meet or enhance.

To enhance the methodological rigor of integrating generative video modeling technologies such as Sora into neurosurgical training, we propose a structured, multiphased approach, informed by both educational theory and technological advancements.

#### Preparation phase

The foundational stage involves the meticulous collection and semantic labeling of video data from a variety of neurosurgical procedures. This dataset, annotated in collaboration with experienced neurosurgeons, aims to highlight key procedural steps, critical decision points, and common pitfalls. Beyond merely informing the AI model, the goal is to craft a multi-dimensional dataset that encapsulates a comprehensive spectrum of neurosurgical scenarios—from the routine to the highly complex. This endeavor leans on principles from cognitive load theory, aiming to optimize the instructional design so that it matches the learner’s cognitive architecture in a way that supports meaningful learning and the development of automaticity in surgical skills.

#### Integration phase

In this phase, Sora will be integrated into existing simulated training environments, which may include virtual reality (VR) and augmented reality (AR) setups already operational in academic medical centers. This integration not only tests the technological feasibility of deploying Sora but also examines its pedagogical effectiveness within established training modules. The integration is designed to support a scaffolded learning approach, where trainees engage progressively with both virtual constructs and AI-enhanced simulations, facilitating a deeper and more cohesive learning experience. This phase reflects the constructivist learning theory, which posits that learners construct knowledge best through active engagement in authentic tasks that simulate real-world processes.

#### Evaluation phase

A pilot study will be conducted to rigorously assess the effectiveness of the integrated training model. This study will involve a cohort of neurosurgical trainees utilizing the system under closely monitored conditions. Performance metrics, established prior to the study, will focus on skill acquisition, accuracy of procedural execution, and decision-making capabilities. The evaluation will employ a mixed-methods approach: qualitative data will be gathered through direct observation and trainee feedback, while quantitative performance analytics will measure the impact of training on various competencies. This phase draws on formative assessment strategies to provide ongoing feedback that can be immediately incorporated into the training process, thereby enhancing learning outcomes. To ensure a thorough assessment of the trainees’ progression and the effectiveness of the integrated training modules, a variety of quantitative and qualitative performance metrics have been established, as outlined in Table 4.

**Table 4:** Overview of quantitative and qualitative performance metrics utilized in the evaluation of neurosurgical trainees

*Quantitative performance metrics*
Technical skill proficiency: Metrics such as accuracy of incisions, precision in suturing, and time to complete specific surgical tasks will be assessed. These are critical in evaluating the trainee’s ability to perform surgical techniques with precision and efficiency
Error rate: This metric involves counting the number of errors (such as incorrect incision sites or improper tool handling) during simulations. It provides a direct measure of the trainee’s technical skill and adaptability to surgical protocols
Procedure completion time: Total time taken to complete a procedure from start to finish. This metric helps gauge the trainee’s fluency and efficiency in conducting surgeries, reflecting their proficiency and speed of integration of surgical knowledge
*Qualitative performance metrics*
Cognitive load assessment: Utilizing subjective measures like the NASA Task Load Index (TLX) to evaluate cognitive load during surgical tasks. This assesses mental, physical, and temporal demands placed on the trainee, providing insights into how effectively they manage complex information under pressure
Decision-making quality: Through structured expert observations and feedback, this metric evaluates the trainee’s ability to make appropriate clinical decisions during simulations. Factors considered include the selection of surgical techniques, intraoperative problem-solving, and adaptability to unexpected complications
Feedback from trainees: Self-reported feedback collected through structured interviews or questionnaires focusing on the trainees’ perceived utility of the AI-enhanced simulations, confidence in performing tasks, and subjective evaluation of their learning experience. This feedback is crucial for adjusting the training modules to better fit trainee needs and expectations
Peer and mentor reviews: Feedback from peers and mentors who observe the trainees during the simulated procedures. This feedback can provide diverse perspectives on the trainee’s performance, highlighting areas of strength and those requiring further improvement
*Integrating metrics into the training evaluation*
The integration of these specific metrics into the evaluation framework involves:
Pre- and post-assessment: Conducting assessments both before and after the training sessions to measure improvements and learning curves
Continuous monitoring: Utilizing sensors and tracking technologies within the simulation environment to gather real-time data on the trainee’s performance
Iterative feedback loop: Incorporating feedback mechanisms where trainees receive immediate, actionable insights after each simulation session, enabling them to adjust and improve in subsequent sessions

This table details the metrics that could be used to assess technical skills, cognitive load, decision-making quality, and feedback mechanisms within Sora-facilitated surgical simulations

#### Validation phase

Upon successful initial evaluation, a larger, multi-center study will be initiated to validate the findings and to examine the scalability and adaptability of the technology across various learning environments and cultural contexts. This phase aims to generalize the effectiveness of Sora in neurosurgical education and provide a robust framework for broader application. The validation study will also explore the longitudinal effects of this training methodology on trainee performance and patient outcomes, adhering to principles of summative assessment and outcome-based education.

This detailed methodological framework not only addresses the technical deployment of cutting-edge AI technologies in medical education but also aligns closely with contemporary educational theories. By fostering an environment where theoretical constructs and technological innovations coalesce, this approach aims to revolutionize neurosurgical training, making it more dynamic, immersive, and fundamentally aligned with the cognitive and practical needs of trainees.

## Results

The study indicates potential benefits of generative video modeling technologies in neurosurgical training, providing immersive simulations that could aid in skill development and enhance exposure to diverse surgical scenarios. However, the technology’s role in standardizing competency assessments remains exploratory. The implementation within neurosurgical education is embryonic, demanding further refinement and empirical validation. These initial observations call for a restrained and methodical examination to conclusively evaluate the impact and utility of such technologies in medical education.

## Discussion

### Innovations and advantages

The proposed integration of Sora into neurosurgical education suggests a departure from conventional training methods toward a more simulation-based approach. Unlike traditional modalities, which typically rely on static media, Sora’s capabilities might allow for the generation of dynamic, interactive simulations tailored to individual learning needs [14]. This approach could potentially extend the range of pathologies and surgical techniques available for educational purposes, although this expansion would depend on the technology’s ability to simulate complex surgical scenarios with high fidelity [15]. The effectiveness of such simulations in actually enhancing surgical proficiency remains to be rigorously tested and verified through empirical studies [3].

### Technical considerations and accessibility

Integrating Sora into neurosurgical training requires addressing several technical challenges, including the creation of a comprehensive dataset of annotated neurosurgical procedures. The development of such a dataset and an effective user interface for interactive learning are non-trivial tasks that involve significant resource investment and technical expertise. Accessibility remains a critical issue, ensuring that such advanced technologies become widely accessible necessitates collaborations between academic institutions, technology developers, and funding organizations. The goal of these partnerships would be to support the deployment and adoption of Sora across diverse educational settings, although the extent to which these efforts can be scaled and sustained is still under exploration [16, 17].

### Limitations and challenges

#### Technical challenges

One of the primary technical challenges with Sora lies in its occasional inability to accurately represent the nuances of spatial relationships and dynamic cause-and-effect interactions that are vital in surgical settings [18]. For example, in neurosurgery, the precise removal of a tumor involves complex interactions between the surgical tools, the delicate surrounding tissues, and the tumor itself [19]. Sora-generated simulations might not fully capture the immediate physical consequences of each surgical action, such as subtle shifts in adjacent tissues or the specific pressure applied during an incision. This limitation can lead to a gap in the trainee’s understanding of tactile feedback and spatial awareness, which are critical for successful surgical outcomes [20].

Moreover, the reliance on existing datasets of video data to train such models restricts the diversity of surgical scenarios that can be simulated. Since these models learn and generate content based on the data they are fed, their capacity to produce novel or uncommon surgical scenarios is inherently limited by the scope of their training data [21]. This could result in a training experience that, while extensive, might not cover the breadth of rare or emergent situations that a neurosurgeon could encounter in a real-world setting.

Achieving accuracy and realism in simulations is essential for effective training [22]. Collaboration with expert neurosurgeons to continuously update and verify the content and accuracy of simulations ensures that the training remains relevant and high quality. Integrating haptic feedback technologies can enhance the realism of interactive simulations, helping trainees to better understand and adapt to the tactile aspects of surgery, which are crucial for skill acquisition.

The collection of surgical videos must be comprehensive and varied, extending beyond typical cases to include rare and complex procedures. This broad spectrum approach ensures that trainees are well-prepared for any situation they might encounter in practice. Employing synthetic data generation techniques can further enrich the training datasets without compromising patient privacy or data integrity [23].

Scalability and accessibility of the technology are fundamental to its success. Developing modular, scalable AI solutions that can be tailored and deployed in diverse educational settings—including those with limited resources—is essential [24]. Partnering with global health organizations can aid in the distribution and localized adaptation of the technology, ensuring it reaches a broad audience.

Continuous learning and model updating mechanisms must be implemented to allow the AI to evolve based on new surgical videos and feedback from actual training use. Utilizing cloud-based technologies can facilitate real-time updates and improvements to the AI models, keeping the technology at the cutting edge of both educational needs and technological advancements [25].

#### Ethical challenges

The use of real patient surgical videos for training AI poses significant ethical challenges, primarily concerning patient privacy and consent [26]. The utilization of actual surgical footage must navigate the complex terrain of confidentiality agreements and ethical standards, ensuring that patient identities are anonymized and that the data are used in a manner that respects patient rights and complies with regulatory standards. Moreover, the potential for bias in the datasets—where certain demographics might be overrepresented or underrepresented—raises further ethical questions about the fairness and inclusiveness of the training scenarios generated by these models.

Enhanced data protection is paramount. Advanced encryption and anonymization techniques must be implemented to secure patient data used in training simulations, ensuring compliance with stringent medical data protection regulations such as HIPAA in the USA and GDPR in Europe [27]. This is not merely a technical requirement but a foundational aspect of maintaining trust and integrity in the use of medical data.

The informed consent process for the use of surgical videos in AI training must be robust. It involves not only obtaining consent but also providing clear, understandable explanations of how data will be used, thus ensuring that patients can make truly informed decisions. This process must be transparent and respectful of patient autonomy, addressing any concerns about data usage comprehensively.

Bias mitigation is another critical area. The datasets used to train such AI technologies must be diversified to include a wide range of patient demographics to prevent the introduction and perpetuation of algorithmic biases. This involves not only the initial composition of datasets but also their regular auditing and updating to ensure they remain representative and fair.

Establishing an ethics board specifically for overseeing the development and application of AI in surgical training is also crucial. This board should include a diverse group of stakeholders such as ethicists, technologists, surgeons, and patient advocates who can provide varied perspectives and ensure that the technology adheres to high ethical standards [28].

#### Empirical study challenges

Empirically assessing the impact of Sora and similar technologies in neurosurgical training introduces additional layers of complexity. Firstly, the design of such studies must contend with the variability in trainee skill levels, the differences in learning environments, and the diverse range of surgical cases that can influence the outcomes of the training. Isolating the effects of the simulation technology from other educational inputs to precisely gauge its effectiveness poses a significant methodological challenge.

Moreover, the longitudinal nature of surgical training, where skills are developed over several years, complicates the ability to conduct short-term studies. Long-term studies, necessary to truly assess the impact of such training technologies, require substantial financial, institutional, and participant commitment, which can be challenging to secure.

Finally, measuring the direct impact of simulation-based training on actual surgical outcomes involves not only educational assessments but also ethical considerations. For instance, tracking the performance of trained surgeons into their professional practice to see the real-world effects of their training involves continuous monitoring and data collection, which raises further privacy and ethical concerns.

In conclusion, while the potential for technologies like Sora to enhance neurosurgical training is significant, realizing this potential requires navigating a complex array of technical challenges, ethical considerations, and empirical research hurdles. Careful development, rigorous testing, and thoughtful implementation, guided by both ethical standards and empirical evidence, are essential to ensure that these technologies truly benefit surgical training without compromising educational integrity or patient safety.

#### Integration of solutions

Forming multidisciplinary teams that include AI developers, surgeons, ethicists, legal experts, and educational specialists ensures that all aspects of the technology’s development are carefully considered. Conducting comprehensive pilot tests to assess both the efficacy and ethical implications of the technology before wider rollout allows for iterative improvements based on real-world feedback.

Maintaining transparency with all stakeholders—including educational institutions, trainees, and the general public—about how the technology works, its benefits, and its limitations is essential for fostering trust and acceptance. This transparency not only helps in managing expectations but also ensures that the technology’s deployment is viewed as both innovative and responsible.

By focusing on these strategies, the discussion on ethical considerations and technical challenges not only deepens but also offers practical and concrete solutions to effectively mitigate these critical issues. This comprehensive approach ensures that the integration of technologies like Sora into neurosurgical training is done ethically, responsibly, and with a focus on long-term sustainability and impact.

### Future directions

As the integration of Sora and comparable generative video modeling technologies into medical education progresses, the necessity for a comprehensive empirical research framework becomes paramount. This research must blend multiple disciplines—educational theory, technology development, and clinical practice—to rigorously test the underlying hypotheses and advance our understanding of these tools. The objective is not only to validate the effectiveness of these models but also to refine their application, ensuring they achieve the high standards necessary for training in complex domains such as neurosurgery.

A pivotal direction for future research is the execution of longitudinal studies, which track the learning outcomes of trainees using these technologies over extended periods. Such studies would assess both short-term proficiency gains and long-term retention of skills, utilizing metrics like procedural accuracy, speed of skill acquisition, and error reduction rates. These data are crucial for understanding how simulation-based learning stacks up against traditional hands-on training, especially concerning long-term skill retention and clinical effectiveness. Concurrently, randomized controlled trials (RCTs) should be employed to provide a robust comparison between traditional training methods and those augmented by simulation technologies. By randomizing trainees into different training modalities, these trials can isolate the effects of the technology from other educational inputs and deliver solid evidence of its impact on various surgical competencies.

Further, the ongoing refinement of the technology itself requires continuous cycles of assessment, feedback incorporation, and reassessment. Research focused on the accuracy and realism of the simulations, utilizing advanced metrics such as motion tracking and biometric feedback, will be essential. These studies not only aid in technological enhancement but also ensure the simulations remain as lifelike and educationally valuable as possible. This task calls for interdisciplinary collaboration, combining the expertise of AI technologists, neurosurgeons, educational psychologists, and ethicists to optimize the educational utility of these tools while adhering to ethical standards. Such collaborations could explore innovative methods for data anonymization, bias reduction, and the creation of diverse, ethically sourced training datasets.

Lastly, exploring the implementation and scalability of these technologies across varied educational settings is critical. This includes assessing the economic costs, infrastructural demands, and training required to adopt these technologies in different geographical and institutional contexts. Such research will help determine the feasibility of wide-scale implementation and identify any potential barriers to adoption.

Together, these research endeavors—encompassing longitudinal outcome studies, controlled trials, technological refinement, interdisciplinary collaborative studies, and implementation research—form a robust agenda aimed at not only validating the effectiveness of generative video modeling technologies but also ensuring their appropriate integration into neurosurgical curricula. This comprehensive approach will crucially dictate the future pathways for employing these advanced educational tools, ensuring they meet evolving medical educational needs.

## Conclusion

The conceptual application of Sora in neurosurgical training presents an innovative, albeit currently hypothetical, approach to enhancing surgical education. By offering dynamic, high-fidelity simulations of surgical procedures, this technology could significantly augment traditional training methodologies, providing a risk-free, immersive learning environment. However, realizing this potential will require overcoming technical, ethical, and practical challenges. As the field of generative video modeling advances, its integration into medical education holds the promise of revolutionizing how future surgeons are trained, pending rigorous validation and thoughtful implementation.

## Data Availability

All data analyzed are contained within the manuscript. No additional data were generated during the course of this study. All relevant results and evidence supporting the conclusions are available to readers, and no supplementary data are required.
